# Leaving care and mental health: outcomes for children in out-of-home care during the transition to adulthood

**DOI:** 10.1186/1478-4505-8-10

**Published:** 2010-05-12

**Authors:** Jane Akister, Matt Owens, Ian M Goodyer

**Affiliations:** 1 Department of Social Work and Social Policy, Anglia Ruskin University, Webb Building, East Road, Cambridge CB1 1PT, UK; 2 Department of Psychiatry, Cambridge University, Douglas House, 18b Trumpington Road, Cambridge CB2 8AH, UK

## Abstract

There were 59,500 Children in out-of-home care in England in 2008. Research into this population points to poor health and quality of life outcomes over the transition to adult independence. This undesirable outcome applies to mental health, education and employment. This lack of wellbeing for the individual is a burden for health and social care services, suggesting limitations in the current policy approaches regarding the transitional pathway from care to adult independence. Although the precise reasons for these poor outcomes are unclear long term outcomes from national birth cohorts suggest that mental health could be a key predictor for subsequent psychosocial adjustment.

Researching the wellbeing of children in out-of-home care has proven difficult due to the range and complexity of the factors leading to being placed in care and the different methods used internationally for recording information. This paper delineates the estimated prevalence of mental health problems for adolescents in the care system, organisational factors, influencing service provision, and pathways through the transition from adolescence to independent young adult life. The extent to which being taken into care as a child moderates adult wellbeing outcomes remains unknown. Whether the care system enhances, reduces or has a null effect on wellbeing and specifically mental health cannot be determined from the current literature. Nonetheless a substantial proportion of young people display resilience and experience successful quality of life outcomes including mental capital. A current and retrospective study of young people transitioning to adult life is proposed to identify factors that have promoted successful outcomes and which would be used to inform policy developments and future longitudinal studies.

## Introduction

*(Children in voluntary or court ordered out-of-home care are referred to differentially between countries and in the literature. In this paper they will be referred to as Children in Care (CIC))*.

Government data in the UK have consistently demonstrated that CIC are overrepresented in the mental health statistics. For example, long term outcomes from national birth cohorts suggest that mental health could be a key predictor for subsequent psychosocial adjustment [1] and a 2002 report commissioned by the Office for National Statistics on behalf of the Department of Health reported that approximately half (45%) of CIC, and almost three quarters (72%) of those in residential care, were clinically diagnosed with a mental disorder [2]. Qualitatively, CIC also differ from the general population and indeed other clinical populations in terms of their complexity of need. It has been further argued, from Australian studies that, *"Children and youth residing away from their parents in court-ordered care represent one of the most vulnerable and disadvantaged groups in Western society. Their mental health problems are complex and exceptional for a non-clinical population." *[[3], p.345]. Despite such complexities and the high relative risk of mental health problems for young people in care, some nonetheless display a resilience, or mental capital, that allows them to achieve positive outcomes. The definition of a positive outcome can of course be made in *absolute *terms but should also be thought of in *relative *terms considering an estimation of initial status at or prior to entry into care.

Mental capital encompasses a person's cognitive and emotional resources and influences both the contribution that they are able to make to society and their experience of wellbeing. "It [mental capital] includes their cognitive ability, how flexible and efficient they are at learning, and their 'emotional intelligence', such as their social skills and resilience in the face of stress." [[4], p.2]. "Mental wellbeing is a dynamic state in which the individual is able to develop their potential, work productively and creatively, build strong and productive relationships with others, and contribute to their community." [[4], p.2]. The Foresight Report proposes that mental wellbeing is enhanced when a person's individual and social goals are fulfilled and they can achieve a sense of purpose in society[[4], p.2].

While children are taken into out-of-home care to protect them from harm, protection from harm is not by itself sufficient to guarantee wellbeing. In order to understand the effectiveness and successful outcomes of the provision of care, individual, social and economic indicators will need to be examined. Individual outcomes would include whether the young person can make a successful transition to adult independence or to appropriate adult services and will be influenced by the age at which they came into care which predicts the likelihood of being responsive to therapeutic and placement opportunities. Social outcomes will include being in education, training or employment and mental wellbeing. Economic outcomes will include the complex question of whether early intervention is effective in both meeting need and in reducing the need for further services.

In the U.K., government guidelines from Every Child Matters suggest promoting five indicators that reflect mental capital: be healthy; stay safe; enjoy and achieve; make a positive contribution; and achieve economic wellbeing [5]. These indicators could be used to examine outcomes from the care system[5].

This paper delineates what we know about the prevalence of mental health problems of young people in care and those who have left care. It also considers the organisational factors that mitigate as to whether children in the care system are referred to mental health services. The paper is organised through the developmental trajectories of mental health in adolescence, the mental health of children in care, the mental health of young people leaving care and the transitions from adolescent mental health to adult mental health services. Prevalence of mental health problems, pathways through services, problems with research methodology and organisational factors in service provision are considered throughout to underpin the understanding of outcomes for young people in care.

### Mental Health during Adolescence

The adolescent period can be a difficult time for many young people. It is a time of transition from school to further and higher education or employment; from childhood dependency to adulthood responsibility and a time of coping with new expectations, physiology and emotions. Adolescence is also a critical period of vulnerability for developing mental health problems such as depression [6]. It has been estimated that approximately half of all lifetime mental disorders begin in the middle teenage years and three quarters by the mid twenties [7]. In this sense mental disorders are distinct from physical disorders as they have their strongest foothold in youth. Conversely, the risk of mental disorder is lower for those who grow through these early, high risk ages unscathed [8,9]

In the UK, approximately 10% of children 5-16 years old are diagnosed with a mental disorder [10]. Research has consistently shown that mental disorders have a serious impact on quality of life in terms of educational outcomes [11], employment and social life [12], and health related outcomes [13]. In public health terms mental disorders account for a larger proportion of disease burden in terms of numbers affected over the lifespan than cancer or HIV [14]. Adolescence is the period when major mental illnesses (depressions, psychoses, severe anxiety disorders, substance abuse and eating disorders) emerge with the greatest incidence and may become associated with a burgeoning impact of such disorders on the individual and society that may last through the lifespan. Thus, research into the impact of mental illness during the adolescent period and its influences on the health and social care trajectories of young people over the transition into adulthood is indicated. Given that CIC have higher levels of mental health problems, research into this highly vulnerable group is especially pertinent.

There have been a number of agency and governmental initiatives to address the issue of risks to mental health in adolescence [e.g. [15-17]]. These initiatives recognise the importance of ensuring the health and wellbeing of children and young people. For example, the abstract for the World Health Organisation's 2008 document declares: "Children are our investment in tomorrow's society. Their health and the way in which we nurture them through adolescence into adulthood will affect the prosperity and stability of countries in the European Region over the coming decades." [[15], p.2]. Similarly, but with an emphasis on services, Standard 4 of the National Service Framework for Children, Young People and Maternity Services [18] is concerned with growing into adulthood. The standard reads: "All young people [should] have access to age-appropriate services which are responsive to their specific needs as they grow into adulthood." [[18], p.119]. More recently, with the imminent completion of the 10 year National Service Framework for Children, a new consultation document, New Horizons [19], proposes the development of new service models for mental health. Key suggestions, for the adolescent age range, are that mental health care could involve youth mental health services that span the period from teens to early adulthood (i.e. 15-24) or could have specialist transition workers, and jointly agreed protocols between children and adult services.

### Mental Health of Children in Care

"Many children come in and out of care and many of those who remain in care frequently change placements. The prevalence of childhood mental disorders tended to decrease with the length of time in their current placement..... The overall rate fell from 49% of those in their current placement for less than a year to 31% of children in their current placement for at least five years." [2]

Most children who enter the care system, in the UK, are placed short-term and will not remain in the system up to their sixteenth birthday, but will be discharged again to the care of their parents with 70% of children being discharged home within a year [20]. A typical example would be one where, for instance, the parent has a period where they are unable to care for their children due to their own mental health problems and once they are well again can resume their care. For those children who are in long-term care retention rates reflect national policy for placement, including whether adoption is permitted under their jurisdiction, making international comparison difficult. The care population therefore is not a stable group and an assessment of their mental health on entry to care is not routine. Assessing changes in mental health or even the prevalence of difficulties at entry, during or after leaving care is problematic.

#### Prevalence

Dimigen, Del Priore, Butler, Evans, Ferguson and Swan [21] assessed the mental health of children, aged 5-12 (n = 89), coming into care in Glasgow, Scotland. Taking measurements within six weeks of admission to care, more than 30% had elevated levels of mental disorders including conduct disorder, depression, attention deficit hyperactive disorder, autism and anxiety. The most common disorders identified were conduct disorders (28%) and depression (28%). They concluded that the complex needs of these children can only be understood effectively through multidisciplinary discussion and strategic planning.

A fundamental question that arises from this research is whether this prevalence rate is any different from other children living in deprivation in private households. Ford, Vostanis, Meltzer and Goodman [[22]; UK] examined the socio-demographic characteristics and psychopathology, by type of placement, of children cared for by local authorities (CIC; n = 1,453). They compared these children with deprived and non-deprived children living in private homes (n = 10,428). Forty six per cent of CIC had at least one psychiatric diagnosis as compared to 15% of the disadvantaged private household sample and 8.5% of the non-disadvantaged private household sample. Children looked after by the local authority have a higher prevalence of both psychosocial adversity and psychiatric disorder than the most socio-economically deprived children living in private households. The prevalence of psychiatric disorder was particularly high among those living in residential care and amongst those experiencing many changes in placement. The mental health issues identified may have arisen from the circumstances that brought the young people into care or indeed the characteristics of some of the young people may have precipitated their reception into the care system.

Barth, Wildfire and Green[[23]; USA] report on the difficulty of determining whether children who enter the child welfare system are from families who are desperately poor or unfit or whether they are entering the child welfare system in order to access mental health services and placements that would be otherwise unavailable to them. They estimate that up to 20% of CIC may have entered due to lack of mental health services and these children, who do not have an unfit parent, are most likely to be in group care. Pecora [[24]; USA] also makes the case for high quality mental health services by reviewing a number of studies in the U.S.A. which all indicate rates of behavioural problems of up to 50% for children, over the age of 11 years, entering the child welfare system. There has been no systematic assessment of the mental health of children on entry to, or during their time in the care system.

#### Pathways through care

Tarren-Sweeney [[3]; New Zealand] reviewing the research into mental health problems of pre-adolescent children in care also finds that their mental health problems more closely resemble clinic-referred than normative samples. Children in residential care have been found to have more mental health problems than those in family-type foster care, with kinship placements recording the least problems. Most available estimates have used standard caregiver-report rating scales primarily the Child Behavior Checklist (CBCL) and the Rutter scales (see Table S1; additional file 1 for details of design, sample characteristics and measures used in referenced studies).

"Studies have estimated a high prevalence of DSM-III-R and DSM-IV conduct disorder (17-45%), attention-deficit hyperactivity disorder (10-30%), depression (4-26%) among mixed samples of children and youth in foster and residential care (13-16). The variability in estimated rates is probably more a consequence of differences in study design, than in the populations selected." [[3], p.348].

One of the key predictors for risk in this group is the age at entry to care with entry at a younger age into family-type care being protective for subsequent mental health.

#### Age on Entry to the Care System

Children entering care in adolescence appear less responsive to changes in parenting style. Possibly the relational difficulties of late-placed children are more resistant to therapeutic change in spite of a positive environment. Cashmore and Paxman [[25]; Australia, n = 47] found that retrospectively measured perceived security was associated with positive outcomes for young adults after they left care. The perceived or 'felt' security is a non-standardised scale adapted from Schofields' framework of a sense of belonging and felt security [[26]; also see Table S1; additional file 1]. Most studies of the mental health problems amongst children in care are cross sectional, but Tarren-Sweeney [[27]; n = 347] reports on a retrospective and concurrent study which identifies age of reception into care and also the perceived or actual sense of security in their placements as predictive for positive outcomes. Tarren-Sweeney[3] argues that given their exceptional vulnerability and the complex problems of this population that they require an assessment of their mental health at the time of entry into care, together with comprehensive assessments of the children's development, social relationships and wellbeing. There is also, we suggest, a case for measuring cognitive performance and educational attainment/neglect on entry into care as this is an outcome indicator that is frequently commented on when children leave care and may be predictive of successful transitions to adult independence. Without evidence of educational engagement on entry to care we cannot gain any real understanding of effectiveness of the care experience on the child's educational progress.

McAuley and Davis [28] review the well-being and mental health of looked after children in Great Britain and concur with the Tarren-Sweeney's findings in relation to the level of mental health problems in this population and with the spread amongst the types of out-of-home care. It is very hard to discern whether the pathway through care influences outcomes for these children as the comparators are the general population. Thus, for example, educational attainment of CIC is reported to be poor (13% of looked after children attaining 5 or more General Certificate of Education grades A-C as compared to 62% of all children in England), but this gives no indication of whether the experience of being looked after has improved or worsened the educational achievement trajectory that these children were on prior to their reception into care.

#### Organisational Factors

What factors determine the service received or the services that young people are referred to? Guglani, Rushton and Ford [29] have reported high levels of psychopathology and educational difficulties in children who are not in out-of-home care but just have contact with social services, together with limited contact with CAMHS services or special educational provision. They found that parents appear to use social services as a frontline service for emotional and behavioural difficulties but often do not get past the duty desk. They do not meet the threshold criteria for social services, and also do not get identified as possibly needing mental health rather than social care services. The issue of identifying when to bring in expertise from other agencies is also reported by Pentecost and Wood surveying 440 child care social workers concerning their knowledge and perception of attention deficit hyperactivity disorder (ADHD) where approximately 30% of the social workers were unaware of local resources that might support these children and families [30]. Thus, for example, inexperienced practitioners did not know of service protocols in place for children with ADHD and were less convinced of the importance of psychiatric assessment than their more experienced colleagues. This study confirms earlier research into social workers' views of the psychiatric needs of children in foster care, where social workers considered 80% required treatment from child mental health practitioners but only 27% were referred [31]. The reasons given for non-referral included placement instability, belief that the mental health facilities available would fail to meet the child's needs and a lack of local authority resources. Other studies have found a lack of structured assessments of the child in Children's Social Services Departments [32]. The use of standardised instruments such as the Strengths and Difficulties Questionnaire (SDQ) could inform assessment through aiding social workers in the detection of mental health problems and providing evidence for referring on to CAMHS. These studies highlight the ongoing organisational challenge of collaboration between health, social welfare and education providers in the detection and management of children's mental health needs.

### Mental Health of Young People Leaving Care

The transition period from care to independent living is a vulnerable time for CIC with increased levels of self-reported mental health problems recorded [33]. Key predictors of poor mental health in risk studies include older age at entry into care, intellectual disability, placement instability and adverse life events [3]. It has also been argued that cumulative adversity is more important in predicting mental health in CIC than specific types of harm or single harmful events [27]. Dixon [33] and Dixon, Wade, Byford, Weatherley and Lee [34] report on a study of 106 young people leaving care. Good preparation for leaving care, with older teenagers, was associated with more successful transitions. Leaving care early (16 to 17 years of age) was associated with poorer outcomes and these young people tended to display more challenging behaviour (including offending and substance misuse). While a young person's predisposition to health difficulties can affect their ability to cope with the transition from care to independent living, conversely, they also suggest that transition from care itself can adversely affect health and well-being. Poor accommodation and isolation can affect a young person's health and affect their coping strategies. Young people who were happy with where they were living and coping well in accommodation were more likely to view their mental health positively. A strong friendship network, good life skills and social skills appeared important in promoting positive wellbeing after care [33]. Leaving care services can help young people to some extent developing relationships and building self-esteem[35].

Health, both physical and mental in the care leavers group is often overlooked in the preoccupations with higher profile areas of accommodation and employment. Dixon [33] interviewed young care leavers and their leaving care workers and found 12% reported mental health problems at the outset with 24% reporting mental health problems on follow-up 12-15 months later (the author suggests that these figures are probably an underestimate as 42% reported emotional and behavioural difficulties at the outset and there is evidence that significant mental health problems may coexist). The explanation for this rise includes the complex difficulties of the post-care transition.

Are difficulties in transition from care a reflection of difficulties experienced pre- care or during care or are they in response to the new challenges of the transition to independent living? An embedded complexity for CIC with mental health problems is that during the phase of transition from care to independent living, there is the potential for another transition to occur; the transition or otherwise from child to adult mental health services. This often happens at 17 years old.

### Transitions from Child and Adolescent Mental Health Services (CAMHS) to Adult Mental Health Services (AMHS)

#### Organisational factors

There is debate about how mental health services to young adults should be organised. McGorry [[36]; Australia] believes that since mental illness often develops in adolescence or young adulthood that the best way to ensure early treatment is to have a service dedicated to the 15-25 age range. Such a service could encompass developmental expertise as an area which is not well serviced by either child or adult mental health services and expertise and where the age of 18 is a poor boundary for service transition. Birleson [37] agrees that more investment is needed in the prevention and intervention for mental health problems early in life, but argues that if we set aside substance use disorder, that the total rate of psychiatric disorder at each life stage varies little and thus the present design of services for children and adolescents who are legally and socially dependent on their parents with a transition to adult services at the age of legal responsibility is appropriate. He argues for integrating and strengthening current systems. Perhaps the real dilemma is simply that transitions, at whatever stage they are determined, are moments of reassessment when there is a risk that some service users will be deemed not to need the next level of services. While this should be a positive outcome, it can often be very difficult to re-engage rapidly with services, if indicated after such a transition. For CIC the difficulty of negotiating a successful pathway through child and adult mental health services may be compounded given the myriad of other changes that could be occurring in their lives.

The TRACK Study tried to identify factors that may facilitate or impede transitions from CAMHS to AMHS in Greater London [[38]; U.K.]. Forty two of the 65 teams contacted responded to the survey showing that 13 transition protocols were in operation and although the protocols identified the centrality of the service users' involvement in the transition process, none of the protocols specified how service users should be prepared for transition with a major gap being the omission from protocols of how to ensure continuity of care for those young people not accepted by AMHS. The researchers consider that the health and social care needs for this group, who slip through the care net, must become a matter of urgent policy.

The New Horizons consultation document [19] states that, "the transition from youth to adulthood is a time when continuity of care is particularly important; however, it frequently breaks down. This is critical not only for the young person, but also for their parents and family." [[19], p.39]. The TRACK study found that less than 5 per cent of adolescents who made the transition received optimal care at the time [38]. Ringeisen, Casanueva, Urato, and Stambaugh [[39]; USA, n = 616; aged 12-15 at baseline and followed up 5-6 years later] report on a study of young adults who had been investigated for child maltreatment in their adolescence by the child welfare systems. They also found a significant decrease in the use of specialist mental health services in the transition from adolescent to young adulthood, declining from 47.6% at baseline to 14.3% on follow-up. Mental health problems were prevalent among the young adults who were suspected of being maltreated when they were adolescents but less than a quarter of those with mental health problems used out-patient mental health services. (They note that uptake of services is influenced by whether the young adults have health insurance and by their race and ethnicity).

#### Pathways

Transitions from child to adult services are difficult whether these are from mental health services or from the services for looked after children. It should be noted that all young people require support in the transition to independent living. Young people leaving care have fewer resources and less support and tend to face the transition at an earlier age than young people in the general population [25]. If all young people need support through the transition, where can those who have already had experience with mental health and social care systems find such support? And what factors mediate successful transitions?

### Understanding Outcomes for Children in Out-of-Home Care

Research into looked after children has been limited because: many of the young people have had numerous placements, health and social care professionals have used different terminologies, systems of data collection have been poor and there are complex reasons why children enter the care system. It is clear, however that young people leaving out-of-home care are over-represented in unemployment, homelessness, teenage parents, disability, lack of formal qualifications and in the prison population [40]. The contributing factors to outcomes from being looked after are hard to disentangle since the experiences of each young person and reasons for entry to the care system are complex. Nonetheless, many children do experience positive outcomes from the care system and others while not successful by population standards may have improved their trajectory through their experience of care.

#### Methodological considerations

All health and social care provision has limited resource. To determine the effective use of these limited resources, "...we need to know who gets mental health care and for what reasons, the costs of that care, and the effectiveness of treatment as related to intensity of service and restrictiveness of setting in order to chart the future course of mental health care services."[[41], p.12]. The Child and Adolescent Services Assessment (CASA) is a self-report instrument that was developed to establish whether self-report in child populations is as reliable as in adult populations where they can provide a reasonably valid indicator of service use. The CASA, while developed with mental health service use as the main focus, aims to identify the whole range of service use accessed within the public sector, including: health, mental health, substance abuse, social service, education and youth justice. It also examines attitudes towards treatment, out-of-pocket costs for treatment and perceived barriers to service use. Results showed that reports of lifetime service use were as reliable as were reports of service use in the last three months and that children reported restrictive and intrusive services more reliably than services that were provided in their natural environment [41,42]. Another similarly named instrument, the Services Assessment for Children and Adolescents also measures mental health services use and treatments and shows high reliability between parent and child reports [43]. Together these measures could be usefully employed in a detailed study of the service use, mental health and other services, by CIC.

Looking at outcomes from care is complicated by a range of factors, including age at entry to care, reason for entering care, duration in care and that CIC include a group who can be described as reunification failures - where a return to their parents care has failed. While most children enter care to protect them from harm, a review of recent studies in the USA reveals that a sizeable group (18%) enter care because of behavioural problems and that for children aged 11 and over entering care this proportion rises to 50%, with many of these remaining in group rather than family based out-of-home care [[44]; USA].

#### Pathways

The Casey Field Office Mental Health study compared rates of lifetime and past year mental health disorder of youth, aged 14 - 17 in foster care (n = 188) with a group matched by age, race/ethnicity and gender from the general population (n = 7753). This produced interesting findings: in both groups, most youth (64%) had no mental health diagnosis in the past year, but the youth in the foster care were more likely to have at least one lifetime diagnosis (63%) as compared to the general population (46%). The foster care group had significantly higher lifetime and past year rates of conduct disorder (21% compared to 7%), ADHD (15% compared to 4.5%), major depressive disorder (19% compared to 12%) and Post Traumatic Stress Disorder (13% compared to 5%). The population sample had higher rates of lifetime and past year hypomania (4% compared to 0%), lifetime panic disorder (2.5% compared to 0%) and past year social phobia (13% compared to 7%) [45].

Pecora et al. [44] conclude that the life circumstances of those leaving foster care place them at higher risk for emotional, behavioural and substance abuse disorder. However, not all young people leaving care have these problems and they propose that careful screening on entry to and during care is needed in a prospective study to try and understand the incidence, duration and severity of mental health problems. They also point to the need to try and understand for which children foster care is an appropriate placement. Barber, Delfabbro and Cooper [[46]; Australia] found that adolescents with mental health problems or behavioural problems had poor placement stability and psychological adjustment indicating an urgent need for a wider range of alternative care options for the adolescent population. Barber and Delfabbro [47] report that foster care is a failure for children with conduct disorder.

Vinnerljung and Sallnäs [[48]; Sweden] report on a study of 718 young people (age 25 years) who were placed in out-of-home care in their teens (aged 13 - 16 years). As with Barber and Delfabbro [47] they found a difference between young people placed in care for behavioural problems (such as delinquency or seriously disruptive behaviour at school) and those placed for other reasons (such as a history of abuse, neglect or mentally unstable parents). Those placed in care for behavioural reasons, as compared to their age group peers who were not placed into care, had very high rates of premature death (5.4% compared to 0.6%), involvement in crime (75% compared to 16%), hospitalization for mental health problems (30% compared to 1%), teenage parenthood and low educational attainment (67% compared to 10%),. Young people placed for other reasons had better outcomes, for example only 48% had low educational outcomes, but still considerably worse than their age group peers. In Sweden 65% of all children entering care were adolescents with the main reason for admission being behaviour problems (it should be noted that Sweden has very few young offenders in the prison system and they are mainly dealt with through the child welfare system so these data are not directly comparable to most other European countries) [48].

### Young People not in Employment, Education, Training (NEET)

Young people during their time in care and on leaving care may belong to the NEET group. There is a dearth of research on young people who are NEET, although a recent report [49] highlighted that approximately 18% of 16-24 year-olds in England are classified as NEET. The Longitudinal Study of Young People in England found that NEET status was associated with poor educational outcomes and that risk behaviours at 13/14 years old were associated with becoming NEET three years later. These included smoking, trying cannabis, engaging in graffiti and vandalism. Alcohol was the exception which did not discriminate between those who did and did not become NEET [50]. With low educational attainment and high relative levels of mental health difficulties indicated from the research there is likelihood that care leavers will be over-represented in the NEET group. With government keen to engage these young people as stakeholders in society there is increased imperative to research and understand positive outcomes from CIC and develop policy to promote these. Therefore the NEET category should be included in future studies.

### Conclusion: The Scale of the Problem and the Way Forward

Recent government statistics have shown that there were 59,500 looked after children (CIC) in England in 2008 [[51]; Table A1]. The research points to poorer outcomes for CIC as a group. Given the numbers of young people involved there is an imperative to understand how those who achieve successful outcomes manage to do this within the care system. This will involve the nature of the individual, the characteristics of their care experience, the extent of their health and education engagement and the quality of support experienced during the transition from care to independent living.

What are the individual and social indicators for the development of resilience or mental capital and successful outcomes? Those young care leavers who do well in terms of mental capital and wellbeing, progress to higher education, do not enter the crime statistics, and are able to maintain independent living with support. The research to date suggests that early entry into the care system, kinship or family based out-of-home care, stability of placement and a personal sense of security promote these successful outcomes. These components promote, in as yet unknown ways, emotional wellbeing and educational attainment. However, the studies use widely different methodologies (see Table S1; additional file 1) and additional work is required to confirm these indicators and to isolate the preferred pathways through care dependent on the reasons for and age at entry to out-of-home care. For example, although kinship or family based out-of-home care appears to offer the best outcomes for the entire cohort of children in care, there is clear evidence that this approach has limited success with older children with conduct disorder coming into out-of-home care and may actually be contraindicated [44,45]. Thus more specific criteria, indicative of successful outcomes, need to be identified in order to develop policy systems which can maximize mental capital and wellbeing in the interests of the individual and the health and social care systems.

More current, and retrospective catch-up studies of young people who have left the care system, with the aim of identifying factors that have promoted successful outcomes with respect to the Every Child Matters Agenda of being healthy; staying safe; enjoying and achieving; making a positive contribution; and achieving economic wellbeing are needed. This will be able to inform policy developments in respect of best practice for looked after children and identify the nature of further longitudinal studies of leaving care and mental health, informing their design and methodology.

## List of abbreviations

ADHD: Attention Deficit Hyperactivity Disorder; AMH: Adult Mental Health Services; CAMH: Child and Adolescent Mental Health Services; CASA: Child and Adolescent Services Assessment; CIC: Children in out-of-home Care; CLAHRC: Collaboration for Leadership in Applied Health Research and Care; NEET: Young People not in Employment, Education, Training.

## Competing interests

IMG is supported by a Wellcome Trust Programme and Project Grant.

## Authors' contributions

JA, MO and IMG devised the framework and set the scope of the review. The literature search was initially carried out by JA and MO. JA drafted the first full version of the paper. All authors contributed to drafting and re-drafting the paper and read and approved the final version of the manuscript.

## Authors' information

JA is a Reader in Social Work at Anglia Ruskin University on sabbatical to the CLAHRC Child and Adolescent Theme.

MO is Research Associate at Cambridge University, Department of Psychiatry and the CLAHRC Child and Adolescent Theme.

IMG is Professor, of Child and Adolescent Psychiatry, Department of Psychiatry at Cambridge University and CLAHRC Child and Adolescent Theme Lead.

## Supplementary Material

Additional file 1 **Design, sample characteristics and measures used in referenced studies**. This file describes the key characteristics of the empirical studies cited in this paper, including country of origin, study design, characteristics of the sample, the measures used and a comments section. Click here for file
