# Supplementary material for: Leaving care and mental health: outcomes for children in out-of-home care during the transition to adulthood
Source: Health Res Policy Syst. 2010 May 12;8:10. doi: 10.1186/1478-4505-8-10 (PMC2890536; doi:10.1186/1478-4505-8-10)
Supplement: Additional file 1 — Design, sample characteristics and measures used in referenced studies. This file describes the key characteristics of the empirical studies cited in this paper, including country of origin, study design, characteristics of the sample, the measures used and a comments section. [file 1478-4505-8-10-S1.DOCX]

| **Table S1. Design, sample characteristics and measures used in referenced studies** | | | | | | |
| --- | --- | --- | --- | --- | --- | --- |
| **Authors** | **Country** | **Design** | **Sample characteristics** | **Measures of mental health** | **Other measures** | **Comments** |
| **Barber**  **et al. (2001)**  **[46]** | Australia | Longitudinal  (4 months) | *n*=235 (170 at time 2).Children referred for out of home care in South Australia between May 1998 and April 1999. 51.5% male. Age range 4-17. | Child Behaviour Checklist  Social adjustment checklist designed by the first two authors |  | Questions asked to social workers, also surveyed a portion of foster carers to check reliability; answers found to be consistent. |
|  |  |  |  |  |  |  |
| **Cashmore**  **et al. (2006)**  [25] | Australia | Longitudinal  (5 years) | All CIC (*n*=47) leaving care between Sep 1992 to Aug 1993 in New South Wales.38.3% male. Age range 16-23. | ‘Success’ across 7 functional domains (of which mental health was one – ‘reported depression or suicidal ideation’) | No. of placements and time spent (stability)  Time spent living with foster carers after leaving care (continuity)  Perceived emotional security (‘felt’ security)  Level of social and emotional support | Uncertain about reliability of measures – all appear to be custom made for this study (although some were based on others previously used).  Young people were interviewed. |
|  |  |  |  |  |  |  |
| **Dimigen**  **et al. (1999)**  [21] | Scotland | Cross-sectional | Children being taken into care (*n*=70) between August 1996 and June 1997; surveyed within first 6 weeks in care.48.6% male. Age range 5-12. | Devereaux scales of mental disorders |  | Questionnaire completed by ‘carer’ – unsure if this means their new carer? If so, their assessment may lack accuracy as they would have known the child less than 6 weeks. |
|  |  |  |  |  |  |  |
| **Dixon (2008)**  [33] | United Kingdom | Longitudinal (12 months) | Sample of CIC (*n*=106) leaving care in 7 English local authorities.47% male. Age range 16-18. | General Health Questionnaire  Lancashire Quality of Life Profile  Cantril’s Ladder | Custom interview questions re: career, accommodation, health, well-being, risk behaviour and support when leaving care) | Young people were interviewed. |
|  |  |  |  |  |  |  |

| **Dixon**  **et al. (2006)**  [34] | United Kingdom | Longitudinal (10 months) | Young people (*n*=106; 101 at time 2)leaving the care of seven local authorities across England. 47% male. Age range 16-19. | Semi-structured interview with some validated measures including:  General Health Questionnaire  Lancashire Quality of Life Profile  Cantril’s Ladder  Life Satisfaction Scale | Schedule to measure service use and cost relevant data  Information re: leaving care worker (including caseload, salary, etc) | Appropriate methods were used for a small sample with limited statistical power.  Both the young person and their leaving care worker were interviewed. |
| --- | --- | --- | --- | --- | --- | --- |
| **Ford**  **et al. (2007)**  [22] | United Kingdom | Cross-sectional, quasi-experimental | *n*=11881. CIC (*n* = 1453) and private household children; randomly sampled from England, Scotland and Wales.57.4% male. Age range 5-15. | Development and Wellbeing Assessment  Strengths and Difficulties Questionnaire ( |  | Appears very robust in design, due to elements such as large sample size, use of random sampling, use of a comparison group, and use of multiple sources of information (including parents, teachers and children if over 11). |
| **Guglani**  **et al. (2008)**  [29] | United Kingdom | Retrospective, longitudinal (3 years) | *n*=298Representative group samples selected from random community sample of 10 438; CIC excluded.  Sample groups:  1. contact with SS  2. contact with CAMH  3. no service contact but difficulties reported.61.6% male. Age range 5-15. | Development and Wellbeing Assessment  Strengths and Difficulties Questionnaire  General Health Questionnaire  (to parent) | British Picture Vocabulary Scale  British Ability Scales Reading Tests  McMaster Family Assessment Device  10 stressful life events  Children’s Services Interview | All measures relied on parental report. |

| **Meltzer**  **et al. (2003)**  [2] | United Kingdom | Cross-sectional | *n*=1039 Random sample of all children (aged 5+) looked after by local authorities in England in first half of 200. Gender not reported. Age range 5-17. | Development and Wellbeing Assessment  Strengths and Difficulties Questionnaire  Moods and Feelings Questionnaire  Survey covered areas such as help-seeking behaviour, strengths, substance use, and behaviour issues. | Survey covered additional information such as academic ability, friendships, and service use. | Teachers, carers and young people (if 11 or older) were surveyed. |
| --- | --- | --- | --- | --- | --- | --- |
|  |  |  |  |  |  |  |
| **Ringeisen**  **et al. (2009)**  [39] | USA | Longitudinal (over 6 years; 5 waves of data collection) | *n*=616 Random sample of children who entered the USA child welfare system due to an investigation of abuse or neglect. 40.3% male. Age range 12-21. | Composite International Diagnostic Interview Short Form  Sections of Trauma Symptoms Inventory  Adult Self-Report  Sections of the Short-Form Health Survey  Child and Adolescent Services Assessment |  | Caregivers and caseworkers interviewed initially; young people spoken to at final follow-up. |
| **Schofield (2002)**  [26] | United Kingdom | Cross-sectional, retrospective, qualitative only | *n*=40Adults admitted to care before 12 years old and who had spent at least three years with one foster family. 25% male. Age range 18-30. |  | Qualitative interviews | Not specifically looking at mental health outcomes but did discuss concepts relevant to emotional security (belonging, loving, etc). |

|  | | | | | | |
| --- | --- | --- | --- | --- | --- | --- |
| **Tarren-Sweeney (2007)**  [27] | Australia | Retrospective | *n*=347 All children in foster/kinship care in New South Wales at time of study. 50.7% male. Age range 4-11. | Child Behaviour Checklist  Assessment Checklist for Children | Carer-report questionnaire (development and education)  Historical data (care history, maltreatment) | Demographic data taken for both carers and birth parents.  Data taken from child welfare database; also reported by carers (carer assessment of child may lack accuracy if it is a new placement for the child). |
| **Vinnerljung et al. (2008)**  [45] | Sweden | Retrospective, longitudinal (using archival data) | *n*=776.70% of all children aged 13-16 that entered Swedish out-of-home care in 1991; outcomes at age 25 examined. 51.5% male. Age range 13-25. | Outcome measures included hospitalisations for mental health problems at ages 20-24, and whether reason for placement in care was due to behavioural issues. | Outcome measures included death before age 25; criminal convictions at ages 20-24, parenthood before 20, receipt of social assistance, educational attainment, and care history. | Study obtained data from social services databases and did not obtain input from the young people themselves. |
| **White**  **et al. (2007)** | USA | Cross-sectional | *n*=188 Children receiving foster care services at a Casey field office in August to November 2006. 48.9% male. Age range 14-17. | Composite International Diagnostic Interview |  | Research appears to be compiled by the company providing the foster care, indicating a potential conflict of interest. |
